# Supplementary material for: Dynamic transcriptomic profiles of zebrafish gills in response to zinc supplementation
Source: BMC Genomics. 2010 Oct 11;11:553. doi: 10.1186/1471-2164-11-553 (PMC3091702; doi:10.1186/1471-2164-11-553)
Supplement: Additional file 2 — Interactive Direct Interaction Network representing the molecular interactions between zinc, copper, iron, calcium and proteins encoded by transcripts changed by zinc supplementation. Mini web-site containing index.html and hyperlinked pages in subdirectory describing a Direct Interaction Network automatically generated based on curated interactions contained within the proprietary PathwayArchitect database. Ovals represent proteins and the circles symbolize metal ions. Objects are coloured by their abundance in zebrafish at the time-point they were significantly different from the control is a scale from -4 fold (dark green) to +4 fold (dark red). Where significant differences were found at more than one time-point, the colour overlay shows expression at the first instance. Dark blue squares denote 'binding', and light blue squares 'expression'; green squares stand for 'regulation', green diamonds for 'metabolism', and green circles for 'promoter binding'. Arrow heads indicate directionality of the interaction where annotated. All nodes and edges can be further interrogated by selecting the relative area of the image. [file 1471-2164-11-553-S2.zip › PathwayArchitect Zn xs DIN/419446.html]

# REGULATION:

|  |  |
| --- | --- |
| Type | REGULATION |
| Effect | None |


---

|  |  |
| --- | --- |
| Score | 0 |


---

|  |  |
| --- | --- |
| Reference Count | 9 |


---

|  |  |
| --- | --- |
| Mechanism | Unknown |


---

|  |  |
| --- | --- |
| Reference:0 || Sentence | "We have previously demonstrated that calpain is responsible for the cleavage of Bax, a proapoptotic protein, during drug-induced apoptosis of HL-60 cells (Wood, D. E., Thomas, A., Devi, L. A., Berman, Y., Beavis, R." |
| PMID | 10075737 |
| Year | 1999 |
| Species | Human |
| Journal | J Biol Chem |
| RefScore | 1 |
| Source | PArchNLP |
  |
|  |  |


---

|  |  |
| --- | --- |
 Reference:1 || Sentence | "In addition, purified calpain enzymes cleaved Bax in a calcium-dependent manner." |
| PMID | 9764817 |
| Year | 1998 |
| Species | Human |
| Journal | Oncogene |
| RefScore | 1 |
| Source | PArchNLP |
  ||  |  |


---

|  |  |
| --- | --- |
 Reference:2 || Sentence | "Previously we showed that Bax was cleaved by calpain rather than by caspases from full-length 21 kDa to generate a cleavage fragment of 18 kDa." |
| PMID | 10772810 |
| Year | 2000 |
| Species | Human |
| Journal | Exp Cell Res |
| RefScore | 1 |
| Source | PArchNLP |
  ||  |  |


---

|  |  |
| --- | --- |
 Reference:3 || Sentence | "Pharmacological calpain inhibition during spontaneous and Fas receptor-induced neutrophil apoptosis prevented cleavage of Bax into an 18-kDa fragment unable to interact with Bcl-xL." |
| PMID | 14612448 |
| Year | 2003 |
| Species | Human |
| Journal | J Biol Chem |
| RefScore | 1 |
| Source | PArchNLP |
  ||  |  |


---

|  |  |
| --- | --- |
 Reference:4 || Sentence | "These results suggested that Bax cleavage is mediated by calpain, and calpain activation may be a caspase-dependent one." |
| PMID | 12175527 |
| Year | 2002 |
| Species | Human |
| Journal | Cancer Lett |
| RefScore | 1 |
| Source | PArchNLP |
  ||  |  |


---

|  |  |
| --- | --- |
 Reference:5 || Sentence | "Taken together, the chemopreventive effects of Se-MSC may be related in part to the caspase-3 activation, the down-regulation of IAP family proteins, and Bax cleavage mediated by caspase-dependent calpain activation." |
| PMID | 12175527 |
| Year | 2002 |
| Species | Human |
| Journal | Cancer Lett |
| RefScore | 2 |
| Source | PArchNLP |
  ||  |  |


---

|  |  |
| --- | --- |
 Reference:6 || Sentence | "These results suggested that Bax cleavage is mediated by calpain, and calpain activation may be caspase-dependent." |
| PMID | 12469198 |
| Year | 2002 |
| Species | Human |
| Journal | Int J Oncol |
| RefScore | 1 |
| Source | PArchNLP |
  ||  |  |


---

|  |  |
| --- | --- |
 Reference:7 || Sentence | "Taken together, the apoptotic effects of NPPA may be related, in part to the caspase-3 activation, the down-regulation of XIAP, and Bax cleavage mediated by caspase-dependent calpain activation." |
| PMID | 12469198 |
| Year | 2002 |
| Species | Human |
| Journal | Int J Oncol |
| RefScore | 0 |
| Source | PArchNLP |
  ||  |  |


---

|  |  |
| --- | --- |
 Reference:8 || Sentence | "Both the calpain-mediated Bax cleavage activity and the Bax/p18 fragment were found in the mitochondrial membrane-enriched fraction." |
| PMID | 11029754 |
| Year | 2000 |
| Species | Human |
| Journal | J Cell Biochem |
| RefScore | 2 |
| Source | PArchNLP |
  |


---

|  |  |
| --- | --- |
